# NetWalker: a contextual network analysis tool for functional genomics

**DOI:** 10.1186/1471-2164-13-282

**Published:** 2012-06-25

**Authors:** Kakajan Komurov, Serkan Dursun, Serkan Erdin, Prahlad T Ram

**Affiliations:** 1 Division of Experimental Hematology and Cancer Biology, Cincinnati Children’s Hospital Medical Center, Cincinnati, OH, USA; 2 Texas Institute of Biotechnology Education and Research (TIBER), Houston, TX, USA; 3 Department of Molecular and Human Genetics, Baylor College of Medicine, Houston, TX, USA; 4 Department of Systems Biology, University of Texas MD Anderson Cancer Center, Houston, TX, USA

**Keywords:** Biological networks, NetWalker, NetWalk, Network analyses

## Abstract

**Background:**

Functional analyses of genomic data within the context of *a priori* biomolecular networks can give valuable mechanistic insights. However, such analyses are not a trivial task, owing to the complexity of biological networks and lack of computational methods for their effective integration with experimental data.

**Results:**

We developed a software application suite, NetWalker, as a one-stop platform featuring a number of novel holistic (i.e. assesses the whole data distribution without requiring data cutoffs) data integration and analysis methods for network-based comparative interpretations of genome-scale data. The central analysis components, NetWalk and FunWalk, are novel random walk-based network analysis methods that provide unique analysis capabilities to assess the entire data distributions together with network connectivity to prioritize molecular and functional networks, respectively, most highlighted in the supplied data. Extensive inter-operability between the analysis components and with external applications, including R, adds to the flexibility of data analyses. Here, we present a detailed computational analysis of our microarray gene expression data from MCF7 cells treated with lethal and sublethal doses of doxorubicin.

**Conclusion:**

NetWalker, a detailed step-by-step tutorial containing the analyses presented in this paper and a manual are available at the web site http://netwalkersuite.org.

## Background

**Figure 1:**
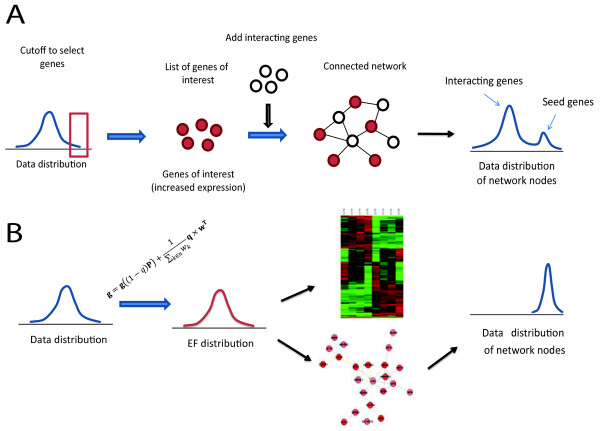
**Comparison of A) list-based methods of network construction and B) NetWalk.** In list-based network construction, interacting genes (open nodes) are added to the network of seed nodes (red) to connect them together. This will generate a single or a number of networks of interest. Distribution of data values of interactor nodes are random. In contrast, NetWalk transforms gene-centric data to interaction-centric data, which can be used for standard statistical analyses (e.g. heatmap analyses) or for dynamic network construction. Data values of nodes constructed through EF values are coherent with input values (see ref.7 for details).

A major goal in *a priori* network-based analyses of genomic data (e.g. gene expression, RNAi screen) is extracting networks of molecular relationships underlying the studied phenotype. Several software tools have been developed for biological network analyses and visualizations, such as Cytoscape [1], BiologicalNetworks [2], VisANT [3], Osprey [4] and BioLayout [5], most of which offer excellent visualization and mapping functions. An important challenge in network-based analyses of data is integration of experimental data with prior knowledge interactions for the retrieval of most relevant biomolecular networks. However, retrieval of most relevant biological networks/pathways associated with the upper or lower end of the data distribution is not a trivial task, mainly because members of a biological pathway do not usually have similar data values (e.g. gene expression change), which necessitates the use of various computational algorithms for finding such networks of genes [1-3,6-9]. Almost all of the existing network-based data analysis methods are so-called list-based network building methods (Figure 1A). These methods use a pre-defined gene list of interest (seed genes) as seeds for iterative network building based on connectivity of non-seed genes with the seed genes. Most software tools, including commercial ones (Ingenuity Pathway Analysis [6]), use this method. Yet others use an enrichment analysis to score pre-defined pathways for enrichment for the seed genes (e.g. MetaCore [10], BiologicalNetworks [2]). A similar approach is usually employed for functional enrichment analyses. We have shown that networks of interest obtained by list-based methods are prone to erroneous inclusion of irrelevant network components [7] (Figure 1). Moreover and importantly, results of list-based analyses are restricted to one or a number of static networks, which are not amenable for further statistical analyses, are not comparable among each other and miss potentially important information contained within genes with sub-threshold data values [11].

In order to provide the research community with a software tool featuring advanced methods for *a priori* network analyses, we developed NetWalker (http://netwalkersuite.org). NetWalker architecture is designed to enable network analyses based on holistic (i.e. no cutoff) integration of experimental data with *a priori* networks and to allow extensive interoperability between analysis components and with external applications. NetWalker features NetWalk [7] and FunWalk, random walk-based analysis methods for prioritization of network interactions and functional processes, respectively, based on assessment of local network connectivity in conjunction with experimental data. Unlike other tools designed for similar purposes, NetWalk and FunWalk allow for interactive comparative analyses of most active networks and functional processes, respectively, between samples. The latter is achieved via Edge Flux and Function Tables, respectively, which give flexibility to the user in querying, analyses and visualizations of networks of most interest. In addition, intuitive inter-operability between analysis and visualization components in NetWalker, as well as with external applications, including R, adds flexibility in data analyses (see Manual in Additional file 1 for more details). In order to demonstrate the use of analysis functionalities in NetWalker for network-based analyses of microarray gene expression data, we have conducted an analysis of our in-house gene expression dataset from doxorubicin responses of p53-positive cells.

## Implementation

**Figure 2:**
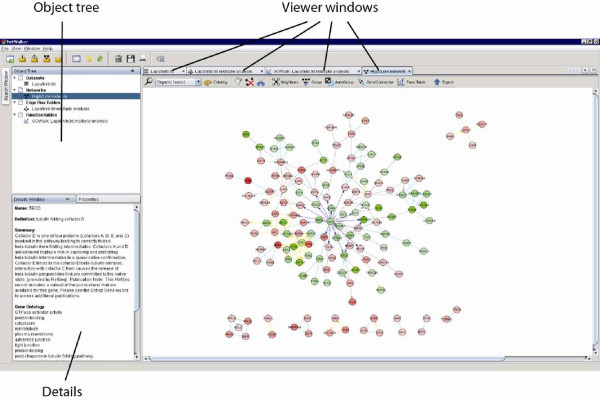
**NetWalker screenshot showing a NetView window with a colored graph.** The Object Tree window shows created DataSet, Function Table, NetView or EF Table objects grouped into branches. The Details window shows annotation details of selected genes or functional terms in networks or in Tables.

**Figure 3:**
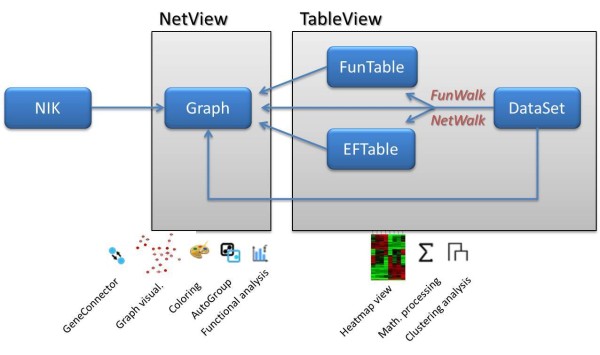
**Object relations in NetWalker.** DataSet is a user-uploaded dataset, which can be analyzed with NetWalk and FunWalk to produce EFTable and FunTable, respectively. DataSet, FunTable and EFTable are handled in TableView and can be queried to create Network Graphs, which are handled/viewed in NetView windows. Graphs can also be created by direct query of NIK, which is loaded at the application startup. TableView offers heatmap, clustering and data table processing functions. NetView offers network view, color mapping, GeneConnector, function analysis and AutoGroup. Data can be easily exchanged between all of these objects by drag and drop or copy/paste.

NetWalker is a software platform specifically designed to allow analyses of functional genomics data within the context of prior networks using whole-population based scoring approaches: NetWalk and FunWalk (see below). NetWalker features functions for data import and processing, network integration and analysis, network visualization, exploration and output (http://netwalkersuite.org). NetWalker was developed in Java, using NetBeans 7.0 (http://www.netbeans.org). A screenshot and general architecture of the software and the relationship between object types are shown in Figures 2 and 3, respectively. A detailed manual for NetWalker is provided in the Additional file 1.

### Software Architecture

In NetWalker, analysis objects are of five types, NetWalker Interactome Knowledgebase (NIK), DataSet, EFTable, FunTable and Graph (Figure 3). The NIK is a pre-compiled knowledgebase of human genes, their functional annotations and their biomolecular interactions. NIK is loaded at the application startup, and it cannot be modified from within the application. The next three objects (DataSet, EFTable and FunTable) are in the form of tables, and Graph represents network views of interest. Tables in NetWalker feature standard functions for statistical manipulation, clustering, heatmap coloring, advanced filtering and network plotting, which give flexibility to the user in the analyses of respective analysis tables. DataSet handles primary datasets, such as gene expression datasets, uploaded by the user. NetWalk is run on selected columns of a DataSet, and generates an EFTable and a FunTable (see next). EFTable is a table of interactions and their scores assigned for each condition that NetWalk was run on. FunTable is a table of functional terms and their scores for each column that FunWalk was run on. Graphs can be derived from any of these three tables by a simple export, or by direct query of the NIK.

### NetWalk and EFTables

The main analysis engine in NetWalker is NetWalk, a random walk-based scoring algorithm of network components based on the assessment of the whole data distribution without requiring any data cutoffs. Briefly, first, the experimental data is integrated with the network to form a transition probability matrix for random walk

(1) $$p_{\mathrm{ij}}=\frac{w_{j}}{\sum {}_{k\in N_{i}}w_{k}}$$

where *p*_*ij*_ is the transition probability from node *i* to node *j*, *w*_*j*_ is the experimental value for node *j*, and *N*_*i*_ is the set of immediate downstream neighbors (undirected edges are considered bidirectional) of node *i*. The probability (*g*) for each node is calculated by the left eigenvector of the modified transition probability matrix:

(2) $$g=g\left(\left(1-q\right)P\right)+\frac{1}{\sum {}_{k\in n}w_{k}}q\times w^{T}$$

where *q* is the restart probability (we use q = 0.01). In NetWalker, we consider interactions, rather than nodes, so we calculate the probability of an interaction *ij* (*e*_*ij*_) as

(3) $$e_{\mathrm{ij}}=g_{i}p_{\mathrm{ij}}$$

**Figure 4:**
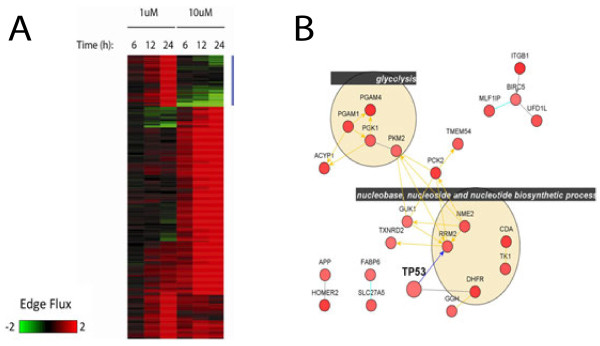
**Edge Flux table analysis of a microarray dataset. A)** Edge Flux heatmap corresponding to interactions that have EF values > 2.25 or > 1.70 in 10 uM–24 h and 1 uM–24 h samples, respectively. Each row in this heatmap corresponds to an interaction, and each value corresponds to Edge Flux values generated by NetWalk. **B)** Plot of the network corresponding to the highlighted rows in A. Nodes are colored by their relative expression in Log2 ratio 10 uM/average condition. Nodes are grouped by selected most enriched functional terms as calculated by Func.Table function in the NetView window.

Finally, the Edge Flux (EF) score of an interaction is calculated by the log-likelihood

(4) $$e_{\mathrm{ij}}=\log\left(\frac{e_{ij\_w}}{e_{ij\_r}}\right)$$

where *e*_*ij_w*_ is the probability of interaction *ij* calculated by using experimental data for *w* in Eqs. 1 and 2, while *e*_*ij_r*_ is that after letting all *w* = 1 in Eqs. 1 and 2 (see ref. for more detailed description). We have shown that networks obtained through NetWalk are more coherent with the input data than those obtained through list-based methods. Moreover, NetWalk results are not limited to one or more static networks, as is the case in most other software, but is a distribution of EF values assigned to each interaction in the network based on the combined assessment of the local connectivity and the corresponding data. Thereby, EF values can be subjected to further standard statistical procedures, such as clustering and heatmap views, for comparative contextual network analyses (see Figure 4 and below). Ability to conduct comparative analyses of networks in the form of EF tables and heatmaps is a fundamentally unique feature in NetWalker, which allows the user to conduct traditional data analyses (such as clustering, t-tests) at an interactome, rather than genome, level. In NetWalker, NetWalk run is performed from within a TableView of a Dataset, and can be run over 1 or more selected data columns. The results are displayed as EF Tables in a TableView, where the interaction details and their scores for each data column is shown. The advanced filtering and further mathematical operations in TableView can be used to create networks of interest or EF heatmaps (see below).

### FunWalk and Function Tables (FunTable)

Functional enrichment analyses are a standard for genomic data. Function enrichment analyses using no-cutoff approaches, exemplified in Gene Set Enrichment Analysis (GSEA), are extremely useful in generating useful insight into the data. However, in addition to its lack of power and other drawbacks [12], GSEA does not account for the connectivity information of genes while calculating enrichment scores. Incorporating connectivity information into the scoring process may provide an additional source of confidence in the relevance of the high-scoring gene sets [13,14] (functional terms). For example, confidence in the relevance of a gene set may be improved if we knew that the genes within the set interact at the molecular level. Although gene set analysis methods, such as GAGE [14], use pre-defined canonical pathways as gene sets in their analyses, they still do not incorporate network connectivity information into their analysis. On the other hand, while GOTEA implemented in VisANT [3] incorporates connectivity information based on network distances of genes in the set, it does not account for experimental data. In order to obtain network-based scores of functional terms in conjunction with the supplied experimental data, we designed FunWalk, an extension of NetWalk, which scores functional terms for their enrichment in the experimental data based on random walk probability scores in Eq. 3. In FunWalk, we aim to prioritize subnetworks with coherent functional annotations whose genes are also over-expressed (or repressed, depending on the goal of analysis) in a given dataset. Therefore, we consider functional annotations of interactions, rather than genes, where the set of functional terms assigned to interaction *ij* is defined as

(5) $$F_{\mathrm{ij}}=F_{i}\cap^{\;}\;F_{j}$$

or the set of common terms of its interacting genes. FunWalk is an extension of NetWalk, such that it calculates the probability of a functional term as the cumulative probability of interactions having the functional term *f:*

(6) $$p\left(f\right)=\sum_{ij\in f}e_{\mathrm{ij}}\;$$

where *e*_*ij*_ is as defined in equation (3). Therefore, the final score for functional term *f* is the log-likelihood:

(7) $$s\left(f\right)=\log\left(\frac{p_{w}\left(f\right)}{p_{r}\left(f\right)}\right)$$

where *p*_*w*_ is the probability of *f* based on experimental data *w*, while *p*_*r*_ is that after setting all *w* = 1. The score *s(f)* can be interpreted as a *relative* visitation probability of interactions defined by the functional term *f* compared to random chance due to network topology and functional set size. Since the lower term in Eq. 7 contains all the bias due to network topology (e.g. more studied genes) and set sizes, the log-likelihood function *s* is controlled for these biases.

Since FunWalk considers functional terms of annotations, rather than genes, it only considers terms that have common annotations across molecular interactions defined in the network. In this way, FunWalk prioritizes *subnetworks* containing common functional annotations that are also over-represented in the data. FunWalk uses NetWalk results to score each functional term for its enrichment in the given dataset. FunWalk results are displayed as Function Tables (FunTable), with each row representing a functional term, and columns show their scores in the given experimental conditions. Any selected rows in a FunTable can be directly exported to a network view in a NetView to view the network interactions associated with the given functional terms.

### NetView, network implementation and functions

NetView windows provide graphical view of networks of interest. NetView contains a number of functions for visual manipulation of the graph, such as different layouts, coloring and functional analyses. For visual representation of network graphs, we have used commercial yFiles library for Java (http://www.yworks.com). The yFiles library offers extensive support for nested graphs, which are important for implementing nested grouping various network layouts. Utilizing yFiles’ support of nested graphs, we have implemented manual and automated grouping of network components.

### NetWalker Interactome Knowledgebase (NIK)

NetWalker uses a pre-compiled knowledgebase of genes, functional terms and biomolecular relationships and is loaded at the application startup.

There are currently 4 different interaction types incorporated into the NIK. These are 1) protein-protein interactions, 2) transcription factor—target interactions, 3) neighboring metabolic reactions, and 4) neighboring interactions from Reactome.

*Protein-protein interactions* were obtained from HPRD (Human protein reference database) [15], BIND (Biomolecular interaction database) [16], MINT [17], BioGRID [18] and IntAct [19]. Directed signaling interactions were obtained from KEGG [20] and NCI Pathway Interaction Database (http://pid.nci.nih.gov/*)*. Interactions from MINT, BioGRID, IntAct and NCI were obtained from Pathway Commons [21].

*Transcription factor—target interactions* were obtained from BIND (queried as protein-dna interactions), Reactome [22] (obtained from Pathway Commons) and NCI Pathway Interaction Database (obtained from Pathway Commons).

*Neighboring metabolic reactions* are assigned to a pair of genes if the product of the reaction catalyzed by one gene is the reactant of the reaction catalyzed by the other. For example, HK2 (Hexokinase II) catalyzes the reaction Glucose + ATP < - > Glucose-6-phosphate + ADP, while GPI (glucose phosphate isomerase) catalyzes the reaction Glucose-6-phosphate - > Fructose-6-phosphate. Since Glucose-6-phosphate is a product of one and the reactant of the other, these two genes are assigned an interaction in the network. See Figure 4B for examples of metabolic interactions (orange interactions). Information on genes and their metabolic reactions were obtained from KEGG, Human Metabolome Database (HMDB) [23]and BiGG [24].

*Neighboring reactions* interactions were obtained from Reactome.

*Functional terms:* Functional annotation of genes from Gene Ontology [25] is used as functional terms for genes in NIK. These are also loaded at the application startup to aid in functional analyses.

The authors will be continuously updating NIK with new interactions from the underlying databases, with new interactions from additional sources and with additional functional annotations of genes. Updated NIK files will be provided at the web site for download.

### ID mapping

Datasets are imported into NetWalker in DataSet Tables (see above and Manual). The column whose values will be used by NetWalker as gene identifiers of rows are set by the user from within DataSet. At this point, NetWalker will automatically match the values in the given column to Gene nodes in the NIK. Currently, supported IDs are Gene Symbols, aliases, Affymetrix probe IDs, Entrez Gene IDs, Refseq, Ensemble, Mouse Genome Database, Rat Genome Database and VEGA IDs.

### R interface

In order to maximize flexibility of analyses in NetWalker, we have implemented an interface with R, a popular statistical programming environment, using network connection. We provide a R workspace file along with the application, which contains currently implemented functions for R-NetWalker interface. Currently, we have implemented functions for exchange of dataset/table and network objects between R and NetWalker. Details on the use of this functionality and sample uses can be found in the Manual.

## License

The software is released with a Creative Commons Attribution-NonCommercial-ShareAlike 3.0 Unported license (CC BY-NC-SA 3.0), which allows for using, modification and sharing of the software and of its components for non-profit purposes.

### Memory requirements and speed

Since NetWalker is using large matrix multiplications for NetWalk and FunWalk, at least 2 GB of memory is required to run NetWalker, although we have successfully been able to run it in systems with less memory. A NetWalk run in NetWalker takes a few seconds per each data column, depending on the sytem. Since EFTables are very large objects (~300,000 EF values per data column), running very large datasets with NetWalk will require large memory (>4 GB) in a 64 bit system running 64 bit JRE.

The yFiles library used in NetWalker allows for visualizations and handling of large networks. We have been able to generate and visualize a network of ~1,000 nodes from an EFTable in under 5 seconds.

### Other

For detailed information on the visual capabilities of NetWalker, along with other functionalities for dataset import, processing, heatmap clustering, GeneConnector and inter-operability with external applications, including R, please see Manual in the Additional file 1. Table 1 contains a summary of comparison of functionalities in NetWalker with other popular software applications.

**Table 1:** Comparison of features in NetWalker, Cytoscape, BiologicalNetworks and VisANT

	**NetWalker**	**Cytoscape**	**BiologicalNetworks**	**VisANT**
**Pre-compiled interactome knowledgebase**	NetWalker Interactome Knowledgebase	No central knowledgebase, can import external interaction sets	IntegromeDB	Predictome
**Dataset import and processing**	Yes	Yes	Yes	No
**Clustering and heatmaps**	Yes	plugin	Yes	No
**Network building with genes of interest**	**GeneConnector**	plugins	Shortest paths, common interactors, filtering	No
**Pre-defined canonical pathways**	No	No	Yes	No
**Whole distribution-based network scoring method**	**NetWalk**	No	No	No
**Unique network integration/analysis method**	**NetWalk, FunWalk, EF Tables, GeneConnector, FunTable**	ActiveModules, other plugins	No	GOTEA, NMEA
**Functional enrichment analysis**	Hypergeometric, **FunWalk**	BiNGO plugin	Fisher’s Exact Probability	GOTEA, NMEA
**Analyses/visualizations of sequence/structure data**	No	No	Yes	No
**Support for non-mammalian species data/networks**	No	Yes	Yes	Yes
**Interoperability with R**	**Yes**	No	No	No

Unique features of NetWalker are in bold.

## Results and discussion

In order to demonstrate the use of functionalities in NetWalker in a real dataset, we undertook an analysis of microarray gene expression data from MCF7 cells before and after treatment with lethal (10 uM) and sublethal (1 uM) doses of chemotherapy drug doxorubicin. We imported the dataset to NetWalker and averaged gene expression values for experimental triplicates for each condition. We normalized gene expression values at each time point to that at the 0 time point to reflect fold change. Then, we ran NetWalk and FunWalk on each of the normalized columns to perform a comparative network analysis of cellular responses to sublethal and lethal doxorubicin doses.

In order to make a heatmap of most significant network interactions in doxorubicin response (EF heatmap), we selected most significant interactions from 1 and 10 uM conditions, and made a clustering heatmap. Figure 4A-B shows the heatmap of most significant interactions associated with increased gene expression in response to low or high doses of doxorubicin and a network corresponding to the highlighted cluster, which represents interactions that are associated with increased expression in high dose but reduced expression in low dose doxorubicin treatment, revealing a bimodal response.

**Figure 5:**
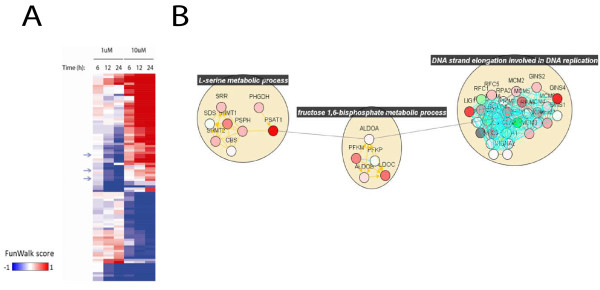
**FunTable analysis of a microarray dataset.A)** Fun Heatmap corresponding to functional terms with most variant FunWalk scores across the 6 conditions. **B)** Plot of the network corresponding to functional terms indicated with arrows in A. Nodes are colored as in Figure 4A, and grouped by their corresponding functional terms.

FunWalk analysis was run together with NetWalk, and a FunTable corresponding to scores of functional terms associated with subnetworks of increased or decreased gene expression was generated. The FunTable was filtered to exclude functional terms with less than 6 interactions and whose level in the GO hierarchy are below 6 (i.e. are too generic GO terms, such as “nucleus”). Then, we identified functional terms with most variant scores across the 6 conditions, and generated a heatmap (Fun Heatmap, Figure 5A). Note a pattern that is very similar to the one in Figure 4A with the EF heatmap, showing a bimodal response of these cells to low and high doses of the DNA damaging agent. Networks corresponding to individual rows can be plotted in a NetView, and a network corresponding to some of the functional terms shown by arrows in Figure 5A is shown in Figure 5B. A detailed step-by-step tutorial on a more detailed analysis of this dataset using various functionalities in NetWalker can be found at the web site http://netwalkersuite.org. The NetWalker workspace environment containing all the analyses presented above can be downloaded from the web site, and loaded in NetWalker.

## Conclusion

In order for a user to be able to analyze his data to extract network models of interest, the software should allow him to a) import and handle the dataset, b) integrate the dataset with a large knowledgebase of biomolecular interactions, c) query networks from the knowledgebase that are most related to his data, d) identify and visualize the networks of interest, e) and visually enhance the network for better representation of the experimental condition. Without any of these components, software will be incomplete, and it will be difficult for a bioinformatically untrained biologist to use it for analysis of his data. For example, VisANT [3], PINA [26], BioLayout [5] and Osprey [4], although offering network construction, management and visualization tools, do not offer functionalities for importing and processing datasets or network integration with user-supplied genomic data, which makes it difficult for biologists to use these tools for network-based data analyses. Cytoscape is a popularly used excellent tool primarily designed for advanced visualizations of networks, but it does not offer content in the form of a knowledgebase. To our knowledge, BiologicalNetworks and NetWalker are the only software platforms that offer all of the functionalities described above. However, NetWalker is the only software that offers efficient holistic (i.e. no cutoff approach) data analysis methods (NetWalk and FunWalk) for comparative network and functional analyses. The design of NetWalker and of the NetWalker Interactome Knowledgebase to emphasize whole-distribution based analysis methods (see Manual for more details) for more flexible data analyses and model building is its most distinguishing feature from other software.

Novel functions can be integrated into existing software applications, such as Cytoscape, instead of developing a stand-alone application. However, Cytoscape is designed more as a visualization tool for biological networks, with some excellent features for visual mapping of data and further visual manipulations. Consequently, Cytoscape is not a database-centric software, like BiologicalNetworks and NetWalker, and the functions it provides, both core and through plugins, mainly concern the networks of interest (usually relatively small networks) uploaded or created by the user. Accordingly, the core API that is used by plugins only provides functions to access the current uploaded networks. In contrast, NetWalker (and BiologicalNetworks) features a pre-compiled knowledgebase of prior information, which is used to query the user-supplied data to extract most relevant networks. In addition, since handling of NetWalk and FunWalk results, their analyses, query and visualizations (EFTable, FunTable and functions therein) are best done with a specialized software architecture, we developed NetWalker as a separate suite to maximize user experience in using these methods. In addition, NetWalk and FunWalk are only pilot methods for the use of biased random walk models in network-based holistic data analyses, and we are currently working on a suite of novel algorithms to be incorporated into NetWalker to enable whole system-based analyses and automated mechanistic model building. Therefore, NetWalker should also be viewed as a novel platform for random walk based holistic network analyses.

## Availability and requirements

NetWalker is available for download for academic use at http://netwalkersuite.org. A Windows and a Mac version have been included. Windows version of NetWalker runs on Windows XP and Windows 7 systems. We have tested the Mac version on a Mac computer with OS X version 10.7. Since NetWalk computations in NetWalker involve many large matrix multiplications, we recommend at least 2 GB of RAM. Most modern processors (Dual Core, Core2 Duo, etc.…) will suffice to run NetWalker with a reasonable performance.

## Competing interests

The authors declare no competing interests.

## Authors’ contributions

KK designed the software and algorithms, performed analyses and wrote the manuscript. SD and SE implemented a clustering algorithm for NetWalker. PTR helped with writing of the manuscript. All authors read and approved final manuscript.

## Supplementary Material

Additional file 1 **NetWalker software manual**. Click here for file
