# Supplementary material for: NetWalker: a contextual network analysis tool for functional genomics
Source: BMC Genomics. 2012 Jun 25;13:282. doi: 10.1186/1471-2164-13-282 (PMC3439272; doi:10.1186/1471-2164-13-282)
Supplement: Additional file 1 — NetWalker software manual. [file 1471-2164-13-282-S1.pdf]

NetWalker Genomic Data Integration Platform

User Guide

Table of Contents

NetWalker Genomic Data Integration Platform 0

General Object Structure and software layout 1

1. NetWalker Interactome Knowledgebase 2

   1.1. Interaction types 2

   1.2 Functional terms 2

2. Handling table objects in TableView 3

   2.1 Dataset import 3

   2.2 TableView functions for data processing 3

3. NetWalk and EFTables 7

4. FunWalk and FunTables 9

5. Graphs and NetView 10

   5.1 NetView functions 10

   5.2 GeneConnector 11

      5.2.1 Successive Addition 11

      5.2.2 Successive Removal 13

6. Interactions with the Clipboard 14

   6.1. Interactions between objects in NetWalker 14

   6.2. Interactions with external applications through the Clipboard 14

7. Search 15

8. R Interface 16

   8.1. Exchanging tables with R 16

   8.2. Exchanging graph objects 16

General Object Structure and software layout

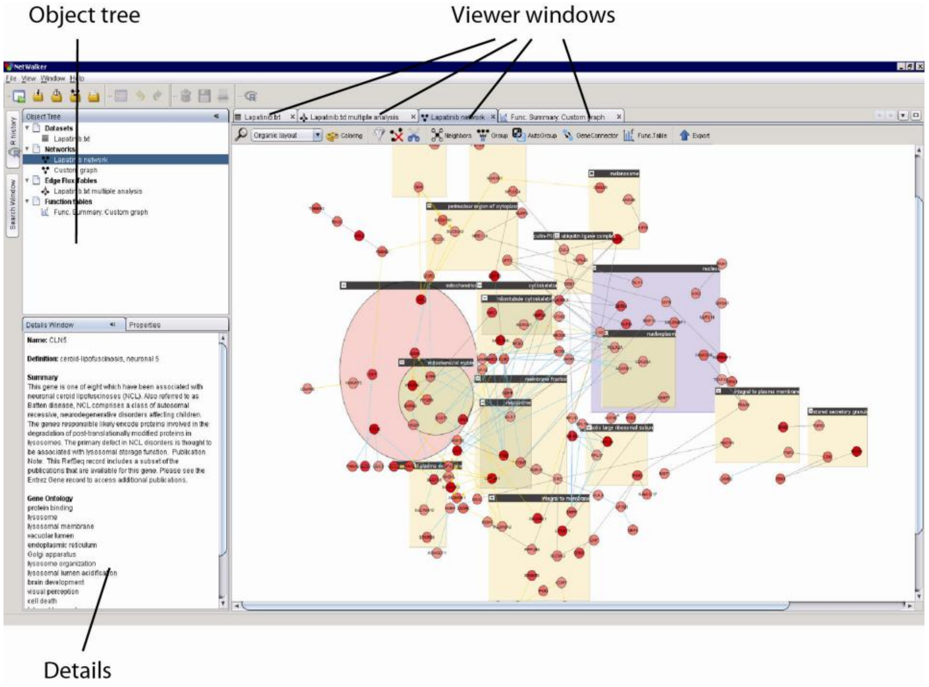

Figure 1. NetWalker main interface.

NetWalker is a desktop application built in Java, utilizing the Netbeans 7.0 platform (www.netbeans.org). The general interface layout is shown in Figure 1. The main toolbar only contains generic functions, such as loading, saving, removing, visualizing the object structures in NetWalker and Undo/Redo functions. Special functionalities pertaining to processing, handling and analyses of data and networks are incorporated into the respective windows for tables and networks for easier access.

NetWalker features five major analysis objects: The NetWalker Interactome Knowledgebase, DataSet, Edge Flux Table (EFTable), Function Table (FunTable) and Graphs. Relationship between these objects is shown in Figure 2. The NetWalker Interactome Knowledgebase is a pre-compiled knowledgebase of human genes, their functional annotations and their biomolecular interactions; and is loaded at the application startup. DataSets are used to represent genomic datasets, EFTables handle results of NetWalk analyses and FunTables are tables of scores generated by FunWalk. The Table objects (DataSet, EFTable and FunTable) are handled in TableView windows (see below). Graphs are objects for visual representations of molecular relationships (edges) between genes (nodes), and are handled in NetView windows (see below). The NIK can be queried by Search window, where the results will be displayed in a NetView window as a network of genes fitting the search criteria. Graphs (or networks, these two terms will be used interchangeably) can also be generated by exporting a set of genes or interactions of interest from a TableView (functional terms from a FunTable, genes from a DataSet or interactions from EFTable). The different windows in NetWalker can be undocked to a floating separate window, docked into a desired location on the application screen or slid into the side bar for customized views.

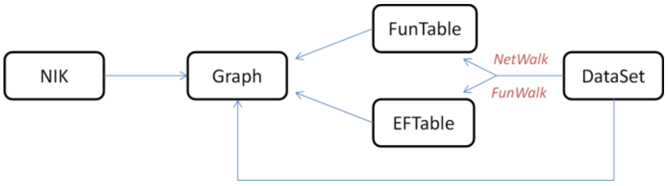

Figure 2. Object relations in NetWalker. DataSet is a user-uploaded dataset, which can be analyzed with NetWalk and FunWalk to produce EFTable and FunTable, respectively. These can be queried to create Network Graphs. Graphs can also be created by direct query of NIK. DataSet, EFTable and FunTable objects are handled in TableView, which allows for them to be analyzed by clustering and heatmaps.

1. NetWalker Interactome Knowledgebase

At the application startup, the user is prompted to select a NIK object out of available ones present within the "Networks" directory. Once the selected NIK is loaded, it will be used for all subsequent analyses within that session and it cannot be edited from within the application. Although users can design their own NIK files according to the criteria below, we provide a pre-designed NIK containing information on genes and their functional interactions. These are summarized below.

1.1. Interaction types

There are currently 4 different interaction types incorporated into the NIK. These are 1) protein-protein interactions, 2) transcription factor – target interactions, 3) neighboring metabolic reactions, and 4) neighboring interactions from Reactome.

Protein-protein interactions were obtained from HPRD (Human protein reference database), BIND (Biomolecular interaction database), MINT, BioGRID and IntAct. Directed signaling interactions were obtained from KEGG and NCI Pathway Interaction Database. Interactions from MINT, BioGRID, IntAct and NCI were obtained from Pathway Commons.

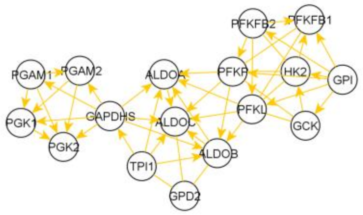

Figure 3. Metabolic reactions from glycolysis. An interaction between two genes indicates consecutive reactions in a metabolic pathway (product of reaction catalyzed by one gene is a reactant for a reaction catalyzed by the other).

Transcription factor – target interactions were obtained from BIND (queried as protein-dna interactions), Reactome (obtained from Pathway Commons) and NCI Pathway Interaction Database (obtained from Pathway Commons).

Neighboring metabolic reactions are assigned to a pair of genes if the product of the reaction catalyzed by one gene is the reactant catalyzed by the other. For example, HK2 (Hexokinase II) catalyzes the reaction Glucose + ATP <-> Glucose-6-phosphate + ADP, while GPI (glucose phosphate isomerase) catalyzes the reaction Glucose-6-phosphate -> Fructose-6-phosphate. Since Glucose-6-phosphate is a product of one and the reactant of the other, these two genes are assigned an interaction in the network. See Figure 3 for a network of glycolytic genes. Information on genes and their metabolic reactions were obtained from KEGG, Human Metabolome Database (HMDB) and BiGG.

Neighboring reactions interactions were obtained from Reactome.

These interaction types capture different aspects of functional relationships between human genes.

1.2 Functional terms

Functional annotation of genes from Gene Ontology is used as functional terms for genes in NIK. These are also loaded the application startup to aid in functional analyses.

2. Handling table objects in TableView

Table objects (DataSet, FunTable and EFTable) are handled in TableView windows. While DataSet objects are original user datasets with experimental data, FunTable and EFTable are derived from data.

2.1 Dataset import

Since the NIK contains information on gene-gene interactions, NetWalker is currently best suited for functional analyses of genomic datasets (e.g. microarray gene expression, aCGH, SNP, genome-wide screens). New datasets can be imported from a file or the clipboard, after which various types of processing can be done on the data columns. NetWalk is performed on data column(s) of a dataset.

Load dataset from file: Datasets can be loaded as tab-delimited (txt) or comma-separated (csv) text files from Menu\Load\Load Dataset or the Load Dataset ( ) button on the main toolbar. The dataset table has to be formatted such that the first row contains column names.

Clipboard: A dataset can be copied from an external spreadsheet application (e.g. MS Excel) and pasted into NetWalker with Ctrl + V or (Edit/Paste menu). However, in order for a Ctrl + V to generate a new table, no window or Object Tree has to be in focus. An option window will appear asking whether the table should be used generation of a new Dataset or a Network.

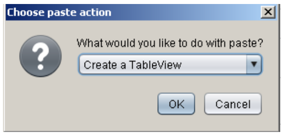

If “Create a TableView” option chosen, the text data in the clipboard will be used to generate a DataSet object within a TableView window. Choosing “Create a Network graph” option will create a new Graph object with matched genes in a NetView window (see below).

Successfully loaded datasets will appear in the Object Tree under **Datasets** and will immediately open in a separate tab window as a TableView. The TableView window contains several functionalities for basic data processing, statistics, plotting, data clustering, heatmaps and network export.

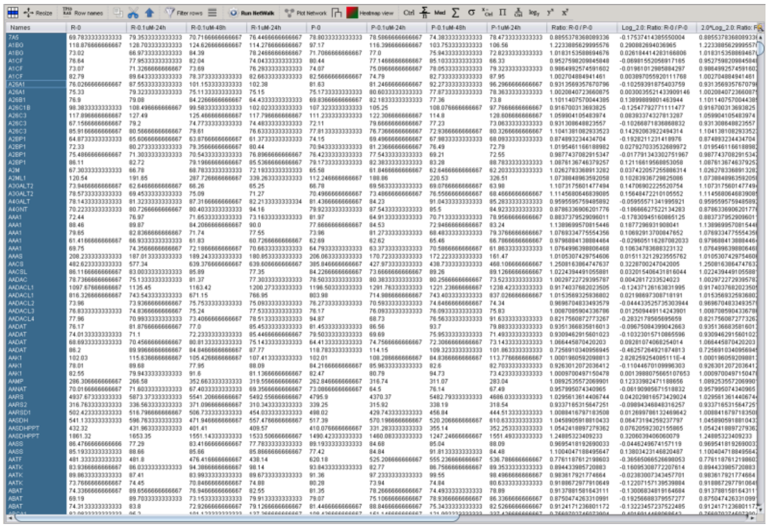

Figure 4. A TableView window with a newly opened dataset.

2.2 TableView functions for data processing

Figure 4 shows TableView functions.

Row/column selection: toggle row or column selection.

Resize rows/columns: lets resize selected columns and rows. This is especially useful

in heatmaps when a whole view of the heatmap is desired.

Set names: when doing network analyses, row identifiers have to be matched to gene names in the NIK. This button allows to set the selected column as the one with gene names. Once gene names are set for a DataSet object, annotation details for the corresponding gene of a row will appear in the Details window upon row selection. This function is missing in the TableView windows holding EFTable and FunTable objects.

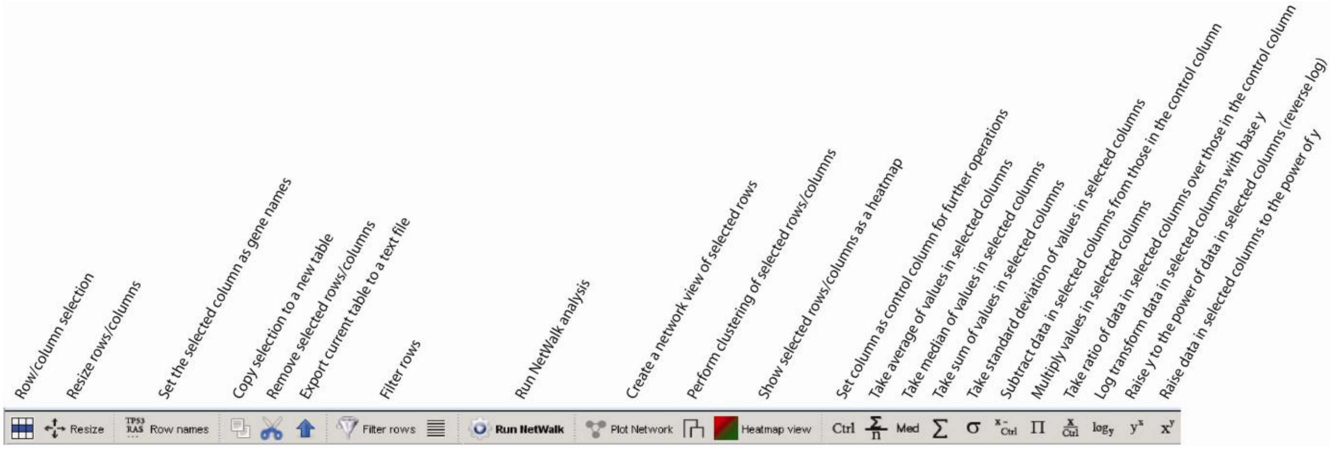

Figure 5. TableView functions and their descriptions.

Gene names are also used as row identifiers during dataset merges (drag and drop one dataset onto another).

Copy selection to a new table: the selected rows and columns will be exported to a new TableView. The newly created TableView object will be of the same type as the source (e.g. if the current object in the TableView is a DataSet, the new object will also be a DataSet).

Remove selected: this removes the selected rows and columns from the TableView.

Export: This will export the current TableView to a tab-delimited text file.

Filter rows: Row filtering is an important function in TableView. This allows to filter out rows for viewing only desired rows. By default, clicking on a column header will sort the rows according to data in that column in the ascending order. Clicking again will sort them in the descending order, and clicking third time will undo all sorting. Additional filters can be done with Filter Rows.

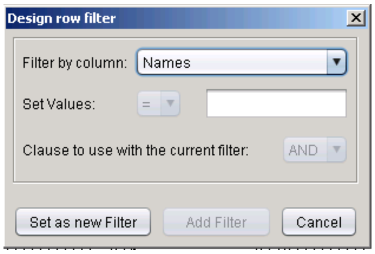

A column should be chosen for filtering, and after that, a value according to which the filtering should be done. For example, if we want to only view rows where gene expression is higher than 2-fold in the sample X, we

would select the column for “sample X” in the above dialog, then select “>” in the drop down menu after “Set Values:”, and put “2” in the text field. When setting the first filter on the TableView, only “Set as new Filter” is available. However, if there is a previously existing filter on the TableView, “Add Filter” also becomes available. In the latter case, the user can choose to set the current filter as the new filter, in which case the previous filtering will be removed and only the new one will be retained, or the newly designed filter can be added on top of the previous filter for a composite filtering. For example, after viewing rows with gene expression >2 in sample X, we decide we also want to see rows where gene expression is higher than 2 in sample Y in the same dataset. In that case, we will press on Row Filter, then select “sample Y” column, and set the filter >2 like we did above. However this time, we put the “Clause to use with the current filter” as “OR”, and we press “Add Filter”. Now, we can see only rows that have gene expression >2 in the sample X OR that are >2 in sample Y. If, we wanted to see rows where gene expression is >2 in sample X AND <-2 in sample Y, we would have selected “AND” in the “Clause…” menu. As can be seen, this function is a useful tool for filtering rows for viewing of rows of interest.

Run NetWalk: for this function, the column for gene names has to be set (otherwise, this button is inactive). Run NetWalk dialog will appear:

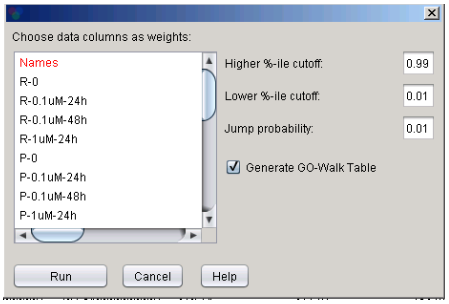

Here, data columns that should be used to run NetWalk are chosen. Ideally, these would be ratio-type data, where data is centered around 1 with non-negative values (e.g. column containing values for ratio of gene expression in sample x over control). Data columns with non-numeric or negative values, which are not suitable for NetWalk, are colored in red. We find that the parameters for NetWalk run, higher and lower percentile cutoffs, should be left at 0.99 and 0.01 for best results (e.g. all data points that are higher than 99th percentile of the total data distribution will be set to the value at 99th percentile value). These cutoff values make sure that the node and edge probabilities in NetWalk are not skewed towards data with outlier values. If “Generate FunWalk Table” is checked, a FunWalk will be run over the same data columns using NetWalk results (see FunWalk and FunTables). Results of the NetWalk analysis will be opened as a new EFTable in a TableView, and that of FunWalk will be opened as a new FunTable in a TableView. This function is missing in TableView windows of FunTables and EFTables.

Plot network: a column for gene names has to be specified for this function. Selected rows will be used to construct a new Graph in a new NetView window. If the current object is a DataSet, genes corresponding to the selected rows will be used. If an EFTable, selected interactions will be used to construct the Graph. If it is a FunTable, interactions/genes corresponding to the selected functional terms will be used to construct the graph object in a NetView.

Clustering: selected rows and columns will be clustered by the given clustering method and the clustering will be used to re-order rows or columns according to the clustering.

Heatmap View: selected rows and columns can be color-coded according the specified color key and color scheme to generate a heatmap. To generate a clustering heatmap, first color-code the desired columns, then perform clustering to re-order them. This will generate a clustering heatmap. Remember that selected rows from this heatmap can be exported to a network view (graph).

Mathematical functions: these functions allow for processing of the data columns. For example, one could use these functions to row-normalize their dataset, calculate average expression of a gene across conditions, perform z-score normalization or calculate log-ratio of fold change over control in a given sample. On each operation, new data will be appended as new column(s) to the table.

3. NetWalk and EFTables

NetWalk is a biased random walk-based method for extracting most relevant *a priori* networks of molecular relationships by simultaneous scoring of network connectivity and the provided data (see Komurov *et al* (2010) PLoS Computational Biology). Unlike most of the existing methods for network extraction (e.g. Ingenuity Pathway Analysis suite, MetaCore), which typically give a set of networks as outputs, NetWalk gives as output a distribution of *EF* (Edge Flux) values: unique values assigned to each interaction in the network scoring their relevance to the given dataset based on simultaneous assessment of the data and local network connectivity. Briefly, Integration of genomic data represented by a vector **w** with the network of interactions between genes (nodes) is performed by representing each interaction (edge) in the network in the form of a transition probability based on the data values (e.g. mRNA expression change, phenotype score from a genetic screen) of nodes within the immediate neighborhood:

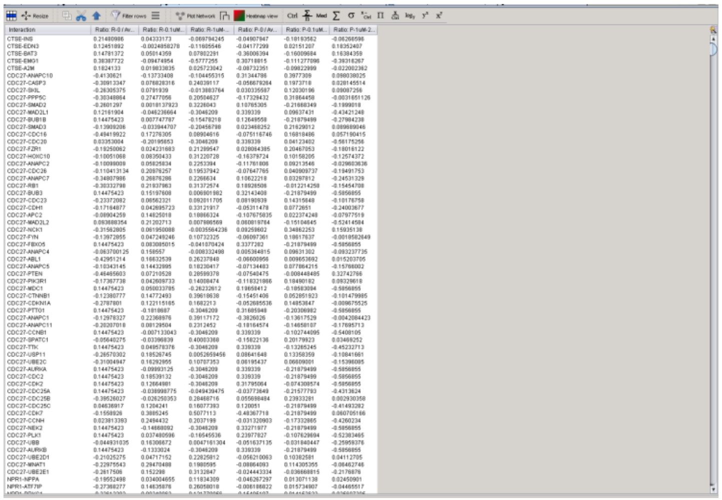

Figure 6. An EFTable in a TableView. First column shows interactions, and each column shows EF values for given samples.

$$p_{ij} = \frac{w_j}{\sum_{k \in N_i} w_k} \qquad (1)$$

where p_{ij} is the transition probability from node i to node j, w_j is the experimental value for node j, and N_i is the set of immediate downstream neighbors (undirected edges are considered bidirectional) of node i. The probability of each node in the network is defined by both the local network connectivity as well the data values of nodes. So at any step k of this "random walk" process, the probability of a node being visited by the random walker is

$$g_i^k = \sum_{j \in n} g_j^{k-1} p_{ji} \qquad (2)$$

where g_i^k is the probability of node i at step k, p_{ji} is the transition probability from node j to node i and N is the set of interacting neighbors of node i. Final probability of a node after infinite random walk ($k = \infty$) based on experimental data w is

$$\mathbf{g} = \mathbf{g}\big((1-q)\mathbf{P}\big) + \frac{1}{\sum_{k \in n} w_k}\mathbf{q} \times \mathbf{w}^{\mathbf{T}} \qquad (3)$$

where q is the restart probability (we use q = 0.01). In NetWalk, we consider interactions, rather than nodes, therefore, we calculate the probability of the interaction between genes i and j as:

$$e_{ij} = g_i p_{ij} \qquad (4)$$

In order to control for topological bias in the network (i.e. nodes with more interactions are likely to be visited more), we calculate the final Edge Flux (EF) score of an interaction as the log likelihood:

$$e_{ij} = \log\left(\frac{g_{i\_w} p_{ij\_w}}{g_{i\_r} p_{ij\_r}}\right) \qquad (5)$$

where the numerator inside log function is the probability of the edge based on experimental values *w*, while the denominator is the probability of the edge based on equal weights to all nodes (i.e. all $w = 1$).

This scoring generates an EF value for each interaction in the network, and allows for flexibility in network construction, as the user can construct networks of any size using different cutoffs of EF values. NetWalk is run on each selected data column in the Run NeWalk dialog (see above), and EF value distribution of the selected samples are displayed as an EFTable in a separate TableView window (Fig.6).

All of the TableView functions described above, except for "Set Names" and "Run NetWalk" are available for EFTables, which allows for their analyses using the mathematical operations, row filtering, clustering and heatmaps. Graphs are generated from selected rows to generate network views in a NetView out of corresponding interactions.

4. FunWalk and FunTables

Functional enrichment analyses are a standard for genomic data. FunWalk is a method for scoring of functional terms based on combined assessment of local network connectivity and genomic data. In FunWalk, we aim to prioritize subnetworks with coherent functional annotations whose genes are also over-expressed (or repressed, depending on the goal of analysis) in a given dataset. Therefore, we consider functional annotations of interactions, rather than genes, where the set of functional terms assigned to interaction *ij* (F_{ij}) is defined as

$$F_{ij} = F_i \cap F_j \qquad (6)$$

or the set of common terms of its interacting genes. FunWalk is an extension of NetWalk, such that it scores a functional term *f* based on random walk visitation probabilities of its member interactions. Briefly, probability of a functional term can be given as the cumulative probabilities of interactions containing the functional term *f*:

$$p(f) = \sum_{ij \in f} e_{ij} \qquad (7)$$

Therefore, the final FunWalk score for the functional term *f* is given by the log-likelihood function:

$$s(f) = \log\left(\frac{p_w(f)}{p_r(f)}\right) \qquad (8)$$

where p_w is the probability of *f* based on experimental data *w*, while p_r is that after setting all $w = 1$. The log-likelihood function allows for controlling of the final score for set sizes of functional terms and topological bias due to more studied genes in the NIK.

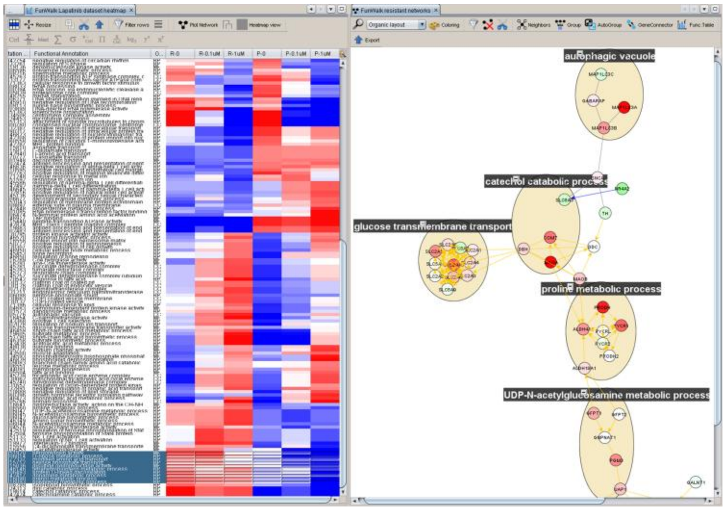

Figure 7. FunWalk analysis of a microarray dataset. The TableView and NetView were arranged side by side. The heatmap on the left shows a heatmap of FunWalk scores with highest variance across 6 conditions. The NetView on the right window shows a network corresponding to the selected GO terms on the bottom of the heatmap, automatically grouped by their corresponding functional terms (see below and Manual for AutoGroup). Nodes in the network are colored by their relative expression in resistant cells.

Since FunWalk considers functional terms of annotations, rather than genes, it only considers terms that have common annotations across molecular interactions defined in the network. In this way, FunWalk prioritizes *subnetworks* containing common functional annotations that are also over-represented in the data. FunWalk uses NetWalk results to score each functional term for its enrichment in the given dataset. FunWalk results are displayed as Function Tables, with each row representing a functional term, and columns show their scores in the given experimental conditions. Any selected rows in a Function Table can be directly exported to a network view in a NetView to view the network interactions associated with the given functional terms. Figure 7 shows an example run of FunWalk.

Selection of a row in a FunTable will display its detailed information in the Details window. A FunTable can also be generated from a Hypergeometric Enrichment analysis from within a NetView window (see below).

5. Graphs and NetView

Graph objects are visual network representations of genes and interactions. Graphs can be generated from any set of genes, interactions or functional terms from within a TableView. In addition, any set of gene names copied into the clipboard from an external application can be pasted into NetWalker to generate a network corresponding to the pasted genes in the NIK. In NetWalker, we make use of the yFiles for Java library to visually represent Graph objects, which provides with state-of-the-art functions for network layout, visualization and hierarchical nesting.

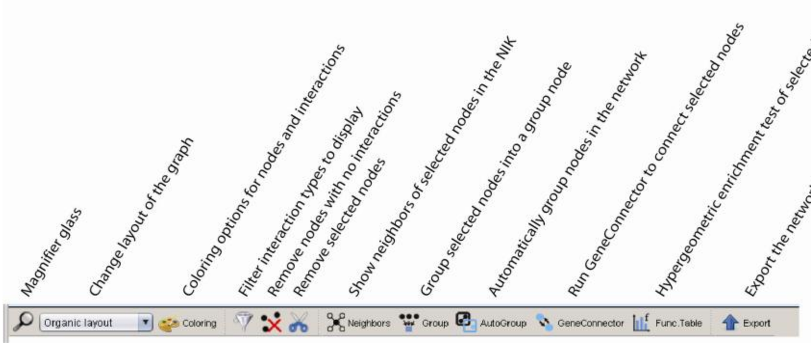

Figure 8. NetView functions and descriptions.

5.1 NetView functions

NetView window features a toolbar with several functions for analyses, editing and visual enhancement of the network graph in view (Figure 8).

Magnifier glass: Selecting this button will turn the magnifier glass feature on, and will allow to magnify local regions of the network view.

Change layout: NetWalker features several layout options supported by the yFiles library. Selecting any of the layouts from the drop-down box will change the layout of the network view accordingly. Organic layout is the most commonly used one. Orthogonal layout may take a while to complete for dense graphs.

Coloring: this will open a drop down panel with coloring options.

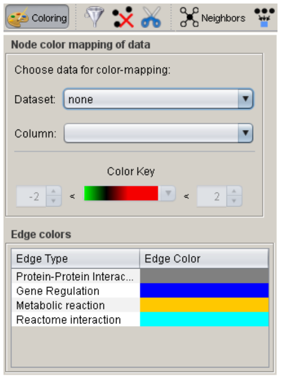

The upper subpanel is for coloring selected nodes based on data values from a dataset. The user chooses a dataset and the column to use for mapping of data to selected nodes. The Gene Names column has to be set in the target dataset for value – node mapping. Color-coding is done according to the Color Key. Interaction types (Edge Type) are shown on the bottom subpanel and their colors in the current NetView. These can be modified to assign different colors to each edge type. The selected coloring will only affect the view in the current NetView and will have no effect on node/edge coloring in other NetView windows.

Filter: this will display a drop-down panel with check boxes where the user can select/unselect interaction types for display.

Remove singletons: this will remove nodes in the graph that have no interactions.

Remove nodes: this will remove selected nodes. Delete key on the keyboard will do the same thing.

Show neighbors: this will fetch neighbors of selected nodes from the NIK and display them on the graph.

Group: this will group selected nodes into a group node. A dialog will ask for a name for the new group.

AutoGroup: this will open a dialog showing all the functional terms assigned to the nodes/genes in the current network graph. The user selects terms he wishes to use to automatically group the network components. If the "hierarchical nesting" box is selected, the algorithm will incorporate the hierarchical structure into the grouping. Otherwise, all functional terms will be treated as separate groups. After clicking on "OK", the user should re-draw the network graph by selecting "Organic Layout" or any other desired layout from the layout drop-down menu.

GeneConnector: this will open a panel with options to run GeneConnector (see below under "GeneConnector").

Functional summary: this will calculate hypergeometric enrichment analyses of functional terms in the selected region of the network graph in view. Results of the functional enrichment analysis will be displayed in a new TableView window as a FunTable.

Export: this will allow for export of the current network view in several formats, including Cytoscape SIF format, a text file, as an image file, to a new NetView window (for selected components), or as an image to the clipboard. With the latter, the image of the current network can be pasted into any image processing software or alike (e.g. a PowerPoint slide, PhotoShop).

5.2 GeneConnector

GeneConnector is a function for finding common networks associated with selected genes/nodes in the network graph. This is equivalent to finding most relevant intermediate genes/nodes that link the selected nodes to each other. One may use GeneConnector when one wishes to find networks associated with a list of genes of interest (e.g. hits from a study). It may be desirable for the user to see how a group of disconnected genes are related to each other in the NIK. For such purposes, there is the GeneConnector functionality implemented within the NetView.

GeneConnector can be run using two connection algorithms: "Successive Addition" and "Successive removal". Successive Addition algorithm adds additional nodes from the NIK onto the network to create an optimally connected network. This method only considers nodes from NIK that have direct interactions with the seed genes.

Successive Removal algorithm works in two stages: first it adds nodes on the shortest path between seed genes to the network. Then, to only retain nodes that *improve* connectivity of the network, it successively removes nodes that are not essential to the connectivity of the network. The second step is required to reduce cluttering of the network.

5.2.1 Successive Addition

Successive Addition adds additional nodes from the NIK to form an *optimally* connected network *G* (see below). There are 3 basic steps in the GeneConnector method. At first, *G* is only composed of the seed genes. Then:

1. Sort all nodes in the NIK according to the sorting function
2. Add the next top gene *a* in the list in (1) onto *G*
3. If the connectivity between the seed genes in *G* have been *improved* according to the connectivity function, retain the gene *a*, otherwise discard, and move onto the next gene
4. Continue till all the genes in the NIK are exhausted

Depending on the sorting function in step 1, and the function for definition of connectivity in step 3, this procedure gives an optimally connected network of genes containing minimum number of genes satisfying the connectivity function. Step 3, where only genes improving connectivity between seed genes are retained, is an essential step ensuring that the final network *G* only contains genes that increase our knowledge about the connectivity of seed genes, which prevents the final network *G* from being overcrowded by redundant intermediate genes and therefore improves visualization and explorability of this network. Because of this filtering process, it can be shown that GeneConnector is superior to existing similar methods in popular commercial network analysis software.

There are two variable functions within the GeneConnector: the node sorting function in step 1, and the connectivity function in step 3.

5.2.1.1 Sorting function

The sorting function sorts all the nodes (genes) in the NIK according to a given criteria of relevance. This is an important step, as nodes will be added onto the resultant network in the order of this relevance. Although multiple different criteria can be defined by which the user may wish to define relevance, 3 different methods have been implemented in NetWalker. These are sorting by 1) Specific connectivity, 2) Data values, and 3) Random walk probability.

<u>Specific connectivity:</u> Specific connectivity $C_{i\text{->}S}$ of a gene *i* to the seed gene list *S* is defined as

$$S_{i\rightarrow S} = \frac{N_{i\rightarrow S}}{N_i} \quad (9)$$

where $N_{i\text{->}S}$ represents the number of interactions of the gene *i* with the seed genes, and N_i shows the total number of interactions of the gene *i*. This option is useful when the user desires to see best-defined connections between the seed genes.

<u>Data values:</u> Alternatively, sorting can be performed based on data values in a dataset, in an ascending or descending order. Depending on the nature of the seed genes and purpose of the user, it may be desirable to prioritize genes based on data. For example, if the seed genes are those that are over-expressed under a certain condition, the user may want to connect these to each other with other genes with increased expression for consistency.

<u>Random walk probability:</u> This is an alternative to Specific Connectivity. NetWalk is run over the Global Network. However, instead of data values for gene weights, all the genes are assigned equal weights of 1 except for the seed genes, which are assigned a value of 10. Genes are then sorted in the order of their visitation probabilities in this random walk process. Genes with highest visitation probabilities are those that are most central to the seed genes in the network. Therefore, this sorting method ensures that genes are sorted according to their centrality to the seed gene list in the Global Network.

5.2.1.2 Connectivity function

This function ensures that only genes that improve the connectivity of the seed genes in the network from the previous step are retained in the network, to prevent over-crowding. Connectivity between seed genes is improved if

$$D_{ij}^{n} < D_{ij}^{n-1} \quad (10)$$

where D_{ij}^{n} is the network distance (geodesic) between genes i and j at the nth iteration; and all i,j are seed genes. Therefore, connectivity between seed genes i and j is improved if the newly added gene reduces their distance in the network. Since we are only interested in new genes that are also directly connected to the seed genes, in theory, the function above is equivalent to

$$D_{ij}^{n-1} > 2 \quad (11)$$

where this time, i,j is over only the seed genes that are directly connected by the newly added gene a (meaning that $D_{ij}^{n} \leq 2$). If we put this equation in the form

$$D_{ij}^{n-1} > k \quad (12)$$

it is obvious that, with increasing k we will achieve more stringent criteria for retaining of new genes in the final network. This k is the *distance threshold* in the connection rules panel.

Overall, GeneConnector is a useful function for analyzing a given list of genes of interest within a network context.

5.2.2 Successive Removal

In Successive Removal method, first, nodes on the shortest path between seed genes will be added to the network *G* to form a connected network. Then, nodes will be successively removed in the reverse order of the sorting function to only retain genes that increase the connectivity information. Basic steps are:

1. Find nodes from NIK that are on the shortest path between all pairs of seed nodes and add to *G*
2. Sort all nodes in *G* in ascending order according to the specified sorting function
3. Remove the bottom gene in the sorting from *G*
4. If connectivity between seed genes in *G* has been affected, don't remove the node and move onto the next bottom gene
5. Continue till all genes in *G* have been exhausted.

In Successive Addition, only genes with direct interactions with seed genes are considered, therefore, seed genes that are far apart in the network may not be connected by Successive Addition. Successive Removal, on the other hand, considers all genes, and therefore, can connect remotely connected seed genes to each other. The second phase in Successive Removal algorithm, which is removing nodes from the network, is important to only retain nodes in *G* that *contribute* to the connectivity of seed genes, which significantly reduces cluttering of the resultant network. Connectivity criteria for retaining a gene in the network are the same as in Successive Addition.

Important Note: Since Successive Removal involves many distance matrix calculations, it is much slower than Successive Addition. Therefore, we recommend using Successive Addition, unless the list of nodes is very small.

6. Interactions with the Clipboard

6.1. Interactions between objects in NetWalker

Objects in NetView and TableView can interact with each other through the clipboard or drag and drop. Dragging a NetView object in the Object Tree onto another NetView allows for comparison of the two corresponding networks. A dialog will ask if the two networks should be added, subtracted from each other or intersected. Same thing can be done by copying a group of selected nodes in a NetView with Ctrl + C, and pasting onto another NetView window with Ctrl + V (Fig.9).

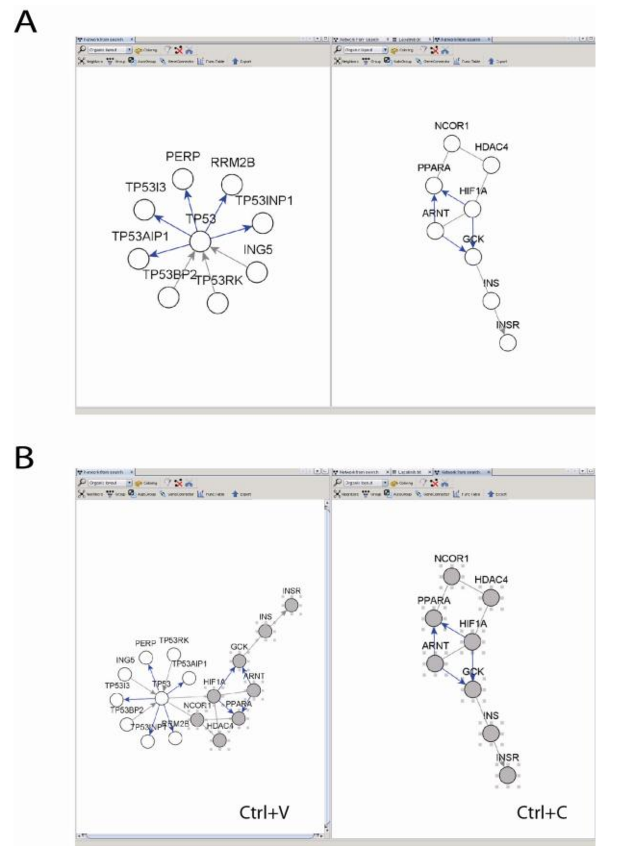

Figure 9. Copy/paste of two networks. A) Two networks in NetView windows arranged side by side. B) The network on right was copied and pasted onto the network on left, choosing A+B as the option.

Similar operations can be done between NetView and TableView objects. If a group of copied nodes from a NetView is pasted onto a TableView (or a NetView is dragged and dropped onto a TableView), a dialog will appear asking whether the pasted nodes should be used to filter the TableView rows, add new rows to the Table or hide rows. In case of filtering rows, only rows that match the node names in their names column will be visible and the rest of rows will be hidden. This is useful when for example one has a gene expression dataset and wishes to see expression profiles in that dataset of a set of genes from another network. In case of adding new rows, this is only useful when the TableView rows are already previously filtered (e.g. by another set of genes from a network), and the user wishes to add these genes to the filter, so that rows with these gene names are also made visible in the TableView. The third option of hiding rows makes rows matching the pasted gene names in their names column will be hidden in the TableView.

A set of rows from a TableView can be copied and pasted onto a NetView to add, subtract or intersect nodes in the recipient NetView with the genes corresponding to the copied rows.

6.2. Interactions with external applications through the Clipboard

All of these copy/paste operations can also be done with any arbitrary copied text from an external application. For example, a set of gene names can be copied from an Excel spreadsheet, and pasted onto a NetView, which will be treated the same way as described above. Similarly, these can be pasted onto a TableView for row filtering as described above. However in addition, pasting into NetWalker with Ctrl+V or View/Paste when no Viewer window (TableView or NetView) is in focus (e.g. click anywhere in the Object Tree window to lose focus of Viewer windows), an option will be presented whether to use the paste to construct a new DataSet TableView or a network. In this way, entire data tables from an Excel spreadsheet or any other external application can be imported into NetWalker by simple copy/paste. Similarly, networks of genes of interest copied/pasted from an external application can be obtained in this way.

7. Search

The Search window can be opened from Windows/Search Window.

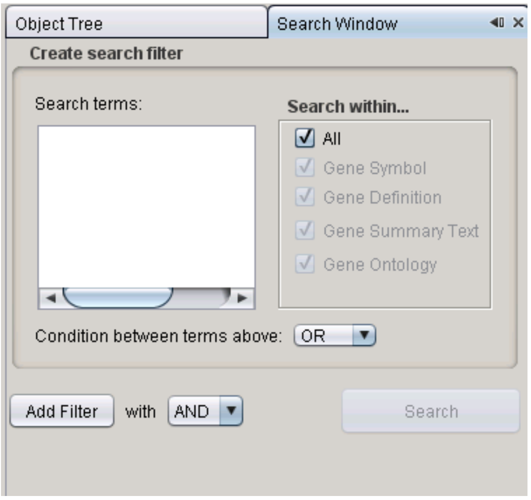

The Search window allows for complex searches by creating Search filters, which can be combined to create complex search phrases. To create a search filter, search terms are entered each on a separate line. "Search within…" shows checkboxes to choose where the search terms should be searched. "Condition between terms above" sets condition between the search terms. Then Add Filter button creates a search filter with the search terms and conditions set in the "Create search filter" panel, and adds it to the current search phrase (not visible). The user can hit Search to search with the existing search filter(s), or create another search filter and add it to the current search phrase with a given clause set using the drop down menu near "Add Filter" button. For example, put "TP53" in search terms and without any modifications press "Add Filter". Then type "glycolysis" in search terms and press "Add Filter" while the combo box near "Add Filter" button is "AND". Hit Search and this will search for genes that have "TP53" AND "glycolysis" in their symbols, definitions, summaries or annotations. The same thing could have been done by creating a single filter with "TP53" and "glycolysis" as separate search terms, but putting "AND" under "Condition between terms above". However, you can think of more complicated search scenarios where creating separate search filters will be necessary.

Results of a search are displayed in a NetView window as a network of genes.

8. R Interface

R Interface provides interface between NetWalker and R GUI. Clicking on R Interface button on the toolbar or under File menu will open the R interface window. The connection with R is through network connection on port 4753. Currently, the R interface allows for exchange of table and graph objects between NetWalker and R through pre-implemented functions. R functions for communicating with NetWalker are found in the R project file found in the installation directory under R, and NetWalker functions for Tables (DataSet, EFTable and FunTable) and graphs are loaded at the application startup and are found in TableView and NetView windows, respectively.

R interface in NetWalker makes use of the svSocket package in R

(http://cran.r-project.org/web/packages/svSocket/index.html), and therefore, this package has to be installed. R server is started from R using NWconnect()

```
>NWconnect()
```

which will start the R server. After this, the user connects to R from within NetWalker using the R interface button under File menu or on the main toolbar.

8.1. Exchanging tables with R

To send a table object from R to NetWalker, use new.table() function in R.

```
>tab <- read.table(…)
>new.table(tab)
```

To send a table object (DataSet, EFTable, FunTable) from NetWalker to R, use “Send table to R” under “R functions” on the TableView toolbar. A dialog will prompt to input a variable name for the new table in R, and a table object corresponding to selected rows and columns in the TableView will be generated under the given variable name.

NOTE: We recommend using the “Send table to R” function in NetWalker with relatively smaller tables (<~1,000 data rows, <~20,000 data points).

8.2. Exchanging graph objects

To send a list of genes of interest from R to NetWalker to generate a corresponding network, use new.graph().

```
>genes <- c(“TP53”, “CDKN1A”, “MDM2”, “ATM”)
>new.graph(genes)
```

Result:

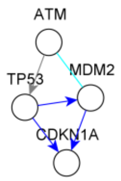

To send a network object from NetWalker to R, use “Send network to R” function under “R Functions” on the NetView toolbar. Again, the user will be prompted to input a variable name to the new network table object in R,

and the network will be sent to R as a list object containing a vector of gene names in the network, and a table of interactions. Send the network above back to R, and name the new variable “p53”.

```
> p53
$genes
[1] "TP53"   "CDKN1A" "MDM2"   "ATM"

$edges
     Source Target   Edge type
[1,] "TP53" "MDM2"   "Gene Regulation"
[2,] "MDM2" "ATM"    "Reactome interaction"
[3,] "ATM"  "TP53"   "Protein-Protein Interaction"
[4,] "MDM2" "CDKN1A" "Gene Regulation"
[5,] "TP53" "CDKN1A" "Gene Regulation"
```

To stop the R interface, hit R interface button under File menu or on the main toolbar again.

We will continue adding more functions to NetWalker to leverage capabilities of R in network analyses in NetWalker.
